# Febrile Seizures and Measles-Containing Vaccines in China: A Self-Controlled Case Series Study

**DOI:** 10.3390/vaccines9101073

**Published:** 2021-09-24

**Authors:** Lu Xu, Ning Li, Liang Zhang, Rui Ma, Ting Fang, Zhike Liu, Siyan Zhan

**Affiliations:** 1 Department of Epidemiology and Biostatistics, School of Public Health, Peking University, Beijing 100191, China; luxu@bjmu.edu.cn (L.X.); zkliu@bjmu.edu.cn (Z.L.); 2 Ningbo Municipal Center for Disease Control and Prevention, Ningbo 315010, China; lin@nbcdc.org.cn (N.L.); zhangl@nbcdc.org.cn (L.Z.); mar@nbcdc.org.cn (R.M.); fangt@nbcdc.org.cn (T.F.); 3 Research Center of Clinical Epidemiology, Peking University Third Hospital, Beijing 100191, China; 4 Center for Intelligent Public Health, Institute for Artificial Intelligence, Peking University, Beijing 100191, China

**Keywords:** febrile seizures, measles-mumps-rubella vaccine, self-controlled case series study, relative incidence

## Abstract

Little is known about the risk of febrile seizures (FS) after vaccination with measles-containing vaccines (MCVs) in middle- and low-income countries. This self-controlled case series study aimed to evaluate the risk of FSs in Chinese children using data from the Ningbo Regional Health Information Platform. The observation period was 0–12 and 13–24 months of age for the MR and MMR vaccines, respectively. The relative incidences (RIs) within 0–6 days, 7–13 days, 14–27 days, and 28–42 days after vaccination with MCVs were estimated. The remaining observation period was the control period. The RIs within 0–6 days, 7–13 days, 14–27 days, and 28–42 days after MR vaccination were 1.11 [95% confidence interval (CI) 0.33 to 3.70], 0.80 (95% CI 0.23 to 2.86), 1.67 (95% CI 0.81 to 3.42), and 1.02 (95% CI 0.49 to 2.14), respectively. The corresponding RIs after MMR vaccination were 0.99 (95% CI 0.56 to 1.75), 1.17 (95% CI 0.68 to 2.01), 0.87 (95% CI 0.54 to 1.39), and 0.85 (95% CI 0.54 to 1.34), respectively. This study suggests that China’s vaccination schedule for MCVs, as suggested by the World Health Organization (WHO) for countries with a high risk of measles mortality and ongoing transmission, does not increase the risk of FSs.

## 1. Introduction

As the most type of seizure disorder among children, particularly those younger than two years old, febrile seizures (FSs) affect approximately 2–5% of children [1]. Infections, especially respiratory tract infections, vaccine administration, etc., are causes of FSs [2].

According to a recent Cochrane systematic review [3], all cohort or self-controlled case series (SCCS) studies published before 2 May 2019, have observed an increased risk of FSs within the risk window of 7–14 days after MMR vaccination [4,5,6,7,8], and no evidence supported an increased risk of FSs in 14 or more days after MMR vaccination [5,6]. In terms of the risk of FSs within one week after MMR vaccination, the results from two cohort studies were controversial [4,5]. Notably, the existing studies on the association between measles-containing vaccines (MCVs) and FSs were all from high-income countries in Europe, America, and Australia, and no such studies came from middle- and low-income countries. However, the vaccination schedule of MCVs varied between high-income countries and middle- and low-income countries. For example, China’s Expanded Program on Immunization (EPI) requires that the first dose of an MCV (i.e., the measles-rubella (MR) vaccine before June 2020 and the MMR vaccine after June 2020) be given at eight months of age, and the second dose of an MCV (i.e., the MMR vaccine) be given at 18 months of age [9], which is in line with the vaccination schedule recommended by the World Health Organization (WHO) for countries with a high risk of measles mortality and ongoing transmission [10]. However, the vaccination schedules of MCVs in most developed countries were in line with the schedule recommended by the WHO for countries with low levels of measles transmission [10], as the first dose of an MCV (i.e., MMR in most developed countries) is given in the second year of life [11,12,13]. Although in China, the first dose of the MMR vaccine is also given in the second year of life when the peak of FSs occurrence exists [14]; the first dose of an MCV is the MR vaccine and not the MMR vaccine in China. Therefore, the risk of FSs after MMR vaccination in the second year of life may be different under China’s vaccination schedule for MCVs. Additionally, the medical sources in most middle- and low-income countries are limited; therefore, the diagnosis, treatment, and outcome of FSs in middle- and low-income countries may be different from those in high-income countries [15].

In China, considering the different vaccination schedules, large population sizes, unique genetic backgrounds, etc., the evidence of vaccine safety should be of great value to global vaccine safety; however, no active surveillance of the association between MCVs and FSs has been conducted in China until now. The Ningbo Regional Health Information Platform (NRHIP) is a united and standardized medical information network in Ningbo, China. In 2016, the NRHIP reached the top level in the Standardization and Maturity Measurement of Regional Health Information Interconnection by the National Health Commission of China [16]. Therefore, this study is the first to use the SCCS design to evaluate the risk of FSs after vaccination with MCVs in Chinese children using the NRHIP.

## 2. Materials and Methods

### 2.1. Data Source

Ningbo, a city in Zhejiang Province, is an important port city on the southeast coast of China. The population in Ningbo was over 8.5 million in 2019. In 2011, the Health Commission of Ningbo began to design and develop the NRHIP to form a systematic and standardized medical information network. By 2015, almost all the health-related activities of more than 87% of Ningbo residents were covered by the NRHIP. The detailed introduction of the NRHIP can be found in a previous study [16]. In this study, the databases of immunization registers and electronic medical records (EMRs) on the NRHIP from 1 January 2016 to 31 December 2019 were used, from which the cases aged 0–24 months were born from 2014 to 2019. The study was approved by the Ethical Review Committee of the Center for Disease Control and Prevention of Ningbo (IRB. No: 202002). The requirement for informed consent was waived.

### 2.2. Study Population

In China, the timing of vaccine administration should be based on the EPI, which requires that children aged eight months to one year should be vaccinated with the MR vaccine and that children aged 18 months to two years should be vaccinated with the MMR vaccine [9,17]. Therefore, this study was restricted to inpatient FS patients aged 0–24 months (0–730 days). Analysis was further restricted to the first inpatient FS episodes of patients aged 0–24 months with vaccination records in the immunization register database. Exclusion criteria due to the violation of the EPI were as follows: children who received ≥two doses of the MR vaccine; children who did not receive the MR vaccine before the MMR vaccine; children who received ≥two doses of the MMR vaccine; children who received the MR vaccine at ages younger than 8 months or older than 12 months; or children who received the MMR vaccine at ages younger than 18 months or older than 24 months. FS cases were identified by using the International Classification of Diseases version 10 (ICD-10) code (i.e., R56) or specific Chinese diagnostic terms of FSs (i.e., “Re Xing Jing Jue” and “Gao Re Jing Jue”).

### 2.3. Case Validation

To evaluate the accuracy of the inpatient FS diagnoses on the NRHIP, the hospital medical charts of 287 inpatient FS patients aged 0–24 months old were reviewed by two pediatricians independently. The judgment criteria were based on three levels of the Brighton diagnostic certainty of generalized convulsive seizure and Brighton Collaboration case definition for fever [18,19]. Any disagreement between the pediatricians was resolved by consulting another senior expert. The positive predictive value was calculated.

### 2.4. Statistical Analysis

The adapted SCCS method for event-dependent exposures (which allows delayed MR or MMR vaccination after the occurrence of FSs) [20] was used to estimate the relative incidences (RIs) of FSs within 0–6 days, 7–13 days, 14–27 days, 28–42 days, and 0–42 days after MR or MMR vaccination [6]. The observation periods for the MR vaccine and MMR vaccine were 0–12 months of age and 13–24 months of age, respectively. The observation period excluding the 0–42 days after receiving an MR or MMR vaccination was defined as an unexposed control period. In the SCCS method, each case acts as its own control by comparing the risk of FS occurrence in exposed periods after MR or MMR vaccination and in unexposed control periods; therefore, time-invariant covariates can be controlled automatically [21]. The potential confounding from age was adjusted by using one-month age bands. Moreover, two sensitivity analyses were conducted: (1) adjustment for age using wider intervals (four-month age groups) and (2) excluding children with a high risk of FSs, i.e., those with a personal history of seizure disorder, infection, neoplasm, central nervous system injury, encephalopathy, or other neurological conditions (Table A1 in Appendix A) [22]; therefore, no children had experienced previous convulsions. Subgroup analysis by sex was performed.

RIs and 95% confidence intervals (CIs) were calculated using conditional Poisson regression. A two-sided *p* value less than 0.05 indicated statistical significance. All statistical analyses were performed with Stata software, version 15.0, and R software, version 3.6.2.

### 2.5. Sample Size

To detect an RI of 1.5 or more within 0 to 42 days after the MCV vaccination, under the assumption of a one-year observation time, 100% vaccine coverage (since vaccination with MCVs on time is considered a societal and obligatory duty in China, coverage with MCVs is high among Chinese children [23]), 80% power and a type 1 alpha level of 0.05, 387 FS episodes were needed.

## 3. Results

### 3.1. Characteristics of the Children with FSs

**Figure 1:**
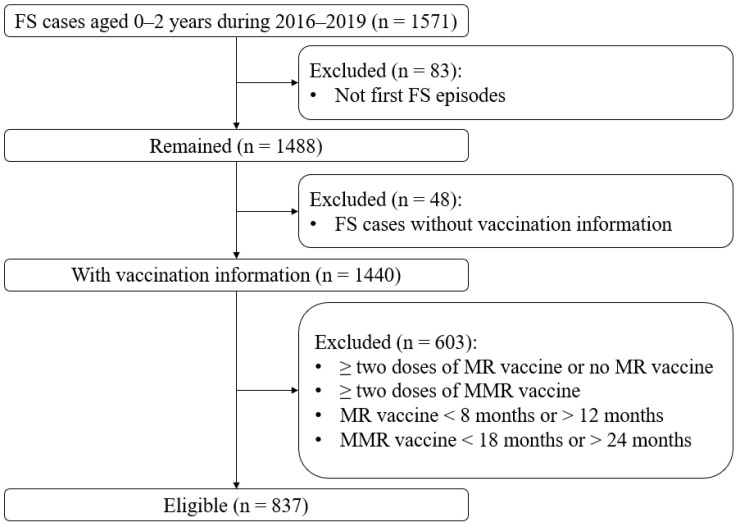
Flowchart of the study participants. Abbreviations: FSs, febrile seizures; MMR, measles-mumps-rubella; MR, measles-rubella.

A total of 837 children (males: 517; females: 320) were enrolled in this study (Figure 1 and Table 1), with a male to female ratio of 1.6. The median onset age of all first FS episodes was 515 days (interquartile range (IQR) 403 to 614).

### 3.2. Case Validation

Among the 287 chart-reviewed FS cases, 260 met level 1 or 2 of the Brighton diagnostic certainty of generalized convulsive seizure and Brighton Collaboration case definition for fever [18,19]. The positive predictive value of the inpatient FS diagnoses among the patients aged 0–24 months on the NRHIP was 91%.

### 3.3. Risk of FSs following MR and MMR Vaccination

The sample size for evaluating the risk of FSs after MR vaccination was 146. The sample size for evaluating the risk of FSs after MMR vaccination was 691. The relative incidences within 0–6 days, 7–13 days, 14–27 days, 28–42 days, and 0–42 days after MR vaccination were 1.11 (95% CI 0.33 to 3.70), 0.80 (95% CI 0.23 to 2.86), 1.67 (95% CI 0.81 to 3.42), 1.02 (95% CI 0.49 to 2.14), and 1.17 (95% CI 0.65 to 2.11), respectively (Table 2). Due to the small sample size (considering that the SCCS model cannot converge), no sensitivity analysis or subgroup analysis was performed to evaluate the risk of FSs after MR vaccination.

The risk of FSs within 0–6 days after MMR vaccination was not higher than the background risk (RI 0.99; 95% CI 0.56 to 1.75) and was similar to the risk of FSs 7–13 days (RI 1.17; 95% CI 0.68 to 2.01), 14–27 days (RI 0.87; 95% CI 0.54 to 1.39) and 28–42 days (RI 0.85; 95% CI 0.54 to 1.34) after MMR vaccination (Table 3). The results of the sensitivity analyses (Table 3) and subgroup analyses (Table 4) by sex also indicated that there was not an increased risk of FSs within the 42 days after MMR vaccination.

## 4. Discussion

This is the first study to assess the risk of FSs after receiving an MR and MMR vaccination in China. No significant association between FSs and vaccination with MCVs in any risk period (0–6 days, 7–13 days, 14–27 days, 28–42 days) among children was found. There was no increased risk of FSs within 7–14 days after MMR vaccination, which is different from the results in European, American, or Australian studies [4,5,6,7,8].

One explanation for the lack of increased FS risk after receiving an MR and MMR vaccination in China may be that the MMR vaccination schedule during the study period in China was different from that in developed countries. In China, the EPI requires that the MR vaccine be given at eight months of age and that the MMR vaccine be given at 18 months of age [9]. However, in the United States, the first dose is given at 12–15 months of age, and the second dose is given at 4–6 years of age [11]. In most European countries, the first dose of the MMR vaccine is given at 12–15 months of age, and the second dose is given at 3–12 years of age [12]. In Australia, the MMR vaccine is recommended to be given at 12 months of age, and the measles, mumps, rubella, and varicella (MMRV) vaccine is recommended to be given at 18 months of age [13]. Therefore, the lack of an increased risk of FSs after receiving an MMR vaccination in the second year of life in Chinese children may be because the vast majority of persons receiving the second dose of MCVs may have already been fully protected by the first dose, which can help neutralize the vaccine virus immediately and completely [24,25,26]. However, we still did not observe an increased risk of FSs after MR vaccination among those aged ≤one year, which may suggest that giving the first dose of an MCV in the first year of life would not increase the risk of FSs. However, the possibility cannot be excluded because the sample size for the MR vaccine was too small to detect the potential risk.

Another explanation may be that the technology of MCV production varies between China and Europe, America, or Australia. First, different virus strains that are used in vaccines may affect the safety of the vaccines [27]. In terms of the measles vaccine strains produced worldwide, most are derived from the Edmonston strain, but the strain used in China was the Shanghai-191 strain. For the rubella vaccine strain, most vaccines used worldwide contain the RA 27/3 strain, but the vaccine produced in China contains the BRD2 strain. Second, the varied excipients may also play a role; for example, most vaccines produced worldwide contain neomycin, except for those produced in China.

Moreover, the misconception of contraindications to immunization is severe in low- or middle-income countries such as China and Africa [28,29], which can lead to healthy vaccinee effects, making it hard to detect adverse events following vaccination [30]). Although to improve vaccination coverage, since the 1980s, the WHO has recommended that some minor illnesses, such as slight fever, mild respiratory infections, and diarrhea, should not be considered as contraindications [31], the problem of false contraindications still cannot be ignored in China. The problem of missed opportunities for immunization caused by false contraindications was more obvious for MCVs than for other vaccines, since the rate of adverse events following immunization caused by MCVs were multiple of other vaccines [28].

The racial or genetic differences between populations in China and the developed countries of Europe or America may also play a role. On the one hand, different immune responses toward MCVs may be taken into consideration, since it was reported that fever is related to a stronger measles antibody response, which is independent of age and the type of MCVs [32]. According to the latest systematic review on MMR vaccine safety and efficacy, the risk ratios (RRs) of the effectiveness of the first dose of the MMR vaccine against measles among children from many cohort studies in European countries were below 0.02 [3], but a study conducted in the Anhui Province of China reported an RR of 0.44 among children aged ≤5 years [17]. Therefore, the immune response toward the MMR vaccine of Chinese children may be lower than that of children from developed countries, which can lead to a lower risk of fever after MMR vaccination, further lowering the risk of FSs among Chinese children. On the other hand, genetic factors have garnered attention recently, as distinct variants may affect the occurrence of FSs after MMR vaccination, or MMR vaccines may be a stimulus to trigger FSs in susceptible individuals [33].

This was the first study in low- and middle-income countries to assess the risk of FSs after vaccination with MCVs by using a population-based regional electronic medical database. However, there were still some limitations. First, due to the lack of medical charts on the NRHIP, we took the visit to the hospital for FSs as the onset time of FSs. However, since FS is an acute disease [34], with the manifestations that can be extremely frightening for parents, parents would take their children to the hospital in a timely manner, and the time interval between FS onset and the visit to the hospital would be short. Second, we cannot distinguish between the primary diagnosis and secondary diagnosis of FSs. Since the primary diagnosis can better reflect the purpose of visiting a doctor, it may, to some extent, affect our results. However, the positive predictive value of the diagnoses of the inpatient FS cases used in this study was up to 91%. Third, we only considered inpatient FS cases, and inpatient FSs may be more severe than outpatient FSs. However, most previous studies also did not include outpatient FS cases [6,7].

## 5. Conclusions

It is reassuring that no increased risk of FSs after MCV vaccination was found among Chinese children, suggesting that China’s vaccination schedule for MCVs (i.e., giving the first dose of an MCV in the first year of life and the second dose in the second year of life), as suggested by the WHO for countries with a high risk of measles mortality and ongoing transmission, does not increase the risk of FSs after vaccination with MCVs.

## Figures and Tables

**Table 1:** Characteristics of the children with FSs included in this study.

Characteristic	Total	Male	Female
No.	837	517	320
Median onset age, days (IQR)	515 (403, 614)	516 (410, 622)	510.5 (400, 606.5)
During the risk period after MR vaccination, No. (%)	33 (3.94)	23 (4.45)	10 (3.13)
During the risk period after MMR vaccination, No. (%)	92 (10.99)	45 (8.70)	47 (14.69)

Abbreviations: FSs, febrile seizures; IQR, interquartile range; MMR, measles-mumps-rubella; MR: measles-rubella.

**Table 2:** Relative incidence of FSs after MR vaccination.

Days since MR Vaccination	No. of FS Cases	Primary Analysis [RI (95% CI, *p*)]
0 to 42 days	33	1.17 (0.65 to 2.11, 0.609)
0 to 6 days	4	1.11 (0.33 to 3.70, 0.871)
7 to 13 days	3	0.80 (0.23 to 2.86, 0.737)
14 to 27 days	14	1.67 (0.81 to 3.42, 0.165)
28 to 42 days	12	1.02 (0.49 to 2.14, 0.954)

Abbreviations: CI, confidence interval; FSs, febrile seizures; MR, measles-rubella; RI, relative incidence.

**Table 3:** Relative incidence of FSs after MMR vaccination.

	No. of FS Cases		Analysis [RI (95% CI, *p*)]		
Days Since MMR Vaccination	Primary Analysis or Sensitivity Analysis One ^a^	Sensitivity Analysis Two ^b^	Primary Analysis	Sensitivity Analysis One	Sensitivity Analysis Two
0 to 42 days	92	90	0.90 (0.64 to 1.27, 0.538)	1.07 (0.81 to 1.41, 0.631)	0.85 (0.60 to 1.20, 0.356)
0 to 6 days	16	16	0.99 (0.56 to 1.75, 0.976)	1.15 (0.68 to 1.93, 0.604)	0.94 (0.53 to 1.66, 0.819)
7 to 13 days	19	19	1.17 (0.68 to 2.01, 0.563)	1.36 (0.83 to 2.23, 0.216)	1.11 (0.64 to 1.90, 0.712)
14 to 27 days	28	26	0.87 (0.54 to 1.39, 0.554)	1.01 (0.66 to 1.53, 0.978)	0.77 (0.47 to 1.24, 0.278)
28 to 42 days	29	29	0.85 (0.54 to 1.34, 0.481)	0.99 (0.66 to 1.48, 0.948)	0.83 (0.53 to 1.31, 0.424)

Abbreviations: CI, confidence interval; FSs, febrile seizures; MMR, measles-mumps-rubella; RI, relative incidence. ^a^ by adjustment for age using wider intervals (four-month age groups); ^b^ by excluding the children who may have a high risk of FSs.

**Table 4:** Relative incidence of FSs after MMR vaccination by sex.

	Male		Female	
Days Since MMR Vaccination	No. of FS Cases	Primary Analysis [RI (95% CI, *p*)]	No. of FS Cases	Primary Analysis [RI (95% CI, *p*)]
0 to 42 days	45	0.66 (0.42 to 1.05, 0.079)	47	1.39 (0.79 to 2.44, 0.247)
0 to 6 days	7	0.64 (0.28 to 1.46, 0.290)	9	1.84 (0.80 to 4.24, 0.153)
7 to 13 days	10	0.92 (0.44 to 1.91, 0.818)	9	1.82 (0.79 to 4.18, 0.159)
14 to 27 days	15	0.70 (0.38 to 1.31, 0.264)	13	1.29 (0.61 to 2.74, 0.505)
28 to 42 days	13	0.59 (0.31 to 1.13, 0.112)	16	1.35 (0.68 to 2.69, 0.390)

Abbreviations: CI, confidence interval; FSs, febrile seizures; MMR, measles-mumps-rubella; RI, relative incidence.

## Data Availability

Due to the Ningbo government’s restrictions, deidentified individual participant data will not be made available.

## Appendix A

**Table A1:** The ICD-10 codes used to identify children who may have a high risk of febrile seizures.

Category of Condition	Condition	ICD-10 Code
Diseases of the nervous system: Inflammatory diseases of the central nervous system	Bacterial meningitis, not classified elsewhere	G00
	Meningitis in bacterial diseases classified elsewhere	G01
	Meningitis in other infectious and parasitic diseases classified elsewhere	G02
	Meningitis due to other and unspecified causes	G03
	Encephalitis, myelitis and encephalomyelitis	G04
	Encephalitis, myelitis and encephalomyelitis in diseases classified elsewhere	G05
	Intracranial and intraspinal abscess and granuloma	G06
	Intracranial and intraspinal abscess and granuloma in diseases classified elsewhere	G07
	Intracranial and intraspinal phlebitis and thrombophlebitis	G08
	Sequelae of inflammatory diseases of the central nervous system	G09
Diseases of the nervous system: Systemic atrophies primarily affecting the central nervous system	Huntington’s disease	G10
	Spinal muscular atrophy and related syndromes	G12
Diseases of the nervous system: Extrapyramidal and movement disorders	Other degenerative diseases of the basal ganglia	G23
	Dystonia	G24
	Other extrapyramidal and movement disorders	G25
	Extrapyramidal and movement disorders in diseases classified elsewhere	G26
Diseases of the nervous system: Other degenerative diseases of the nervous system	Other degenerative diseases of nervous system, not classified elsewhere	G31
	Other degenerative disorders of nervous system in diseases classified elsewhere	G32
Diseases of the nervous system: Episodic and paroxysmal disorders	Epilepsy	G40
Diseases of the nervous system: Other disorders of the nervous system	Disorders of the autonomic nervous system	G90
	Hydrocephalus	G91
	Toxic encephalopathy	G92
	Other disorders of the brain	G93
	Other disorders of the brain in diseases classified elsewhere	G94
	Other disorders of the central nervous system	G96
	Postprocedural disorders of the nervous system, not classified elsewhere	G97
	Other disorders of the nervous system, not classified elsewhere	G98
	Other disorders of the nervous system in diseases classified elsewhere	G99
Metabolic disorders	Sphingolipid metabolism disorders and other lipid storage disorders	E75
Infectious and parasitic diseases	Tuberculosis of the nervous system	A17
	Atypical virus infections of the central nervous system	A81
	Mosquito-borne viral encephalitis	A83
	Tick-borne viral encephalitis	A84
	Other viral encephalitis, not classified elsewhere	A85
	Unspecified viral encephalitis	A86
	Viral meningitis	A87
	Other viral infections of the central nervous system, not classified elsewhere	A88
	Unspecified viral infection of the central nervous system	A89
	Other mosquito-borne viral fevers	A92
	Other arthropod-borne viral fevers, not classified elsewhere	A93
	Unspecified arthropod-borne viral fever	A94
Neoplasms	Malignant neoplasm of the meninges	C70
	Malignant neoplasm of the brain	C71
	Malignant neoplasm of the spinal cord, cranial nerves and other parts of the central nervous system	C72
	Benign neoplasm of the meninges	D32
	Benign neoplasm of the brain and other parts of the central nervous system	D33
Injuries to the head	Fracture of the skull and facial bones	S02
	Intracranial injury	S06
	Crushing injury of the head	S07
	Traumatic amputation of part of the head	S08
	Other and unspecified injuries of the head	S09
